# Patients’ perioperative experiences of an opioid-free versus opioid-based care pathway for laparoscopic bariatric surgery: A qualitative study

**DOI:** 10.1016/j.ijnsa.2024.100201

**Published:** 2024-04-20

**Authors:** Alexander Olausson, Eva Angelini, Birgit Heckemann, Paulin Andréll, Pether Jildenstål, Sven-Egron Thörn, Axel Wolf

**Affiliations:** a Institute of Health and Care Sciences, Sahlgrenska Academy, University of Gothenburg, Sweden; b Sahlgrenska University Hospital/Östra, Department of Anaesthesiology and Intensive Care Medicine, Gothenburg, Sweden; c Department of Anaesthesiology and Intensive Care Medicine, Institute of Clinical Sciences at Sahlgrenska Academy, University of Gothenburg, Sweden; d Department of Anaesthesiology and Intensive Care Medicine/Pain Centre, Sahlgrenska University Hospital, Region Västra Götaland, Gothenburg, Sweden; e Department of Health Sciences, Lund University, Lund, Sweden; f Sahlgrenska University Hospital, Department of Anaesthesiology and Intensive Care Medicine, Gothenburg, Sweden; g Department of Anesthesiology and Intensive Care, Örebro University Hospital and School of Medical Sciences, Örebro University, Örebro, Sweden; h Institute of Nursing and Health Promotion, Oslo Metropolitan University, Oslo, Norway; i Centre for Person-Centred Care (GPCC), University of Gothenburg, Sweden

**Keywords:** Bariatric surgery, Nursing research, Opioid-free anaesthesia, Patient experience, Perioperative care

## Abstract

**Background:**

Despite recent evidence supporting the adoption of opioid-free anaesthetic and analgesic alternatives in the perioperative context, opioid-based regimens remain standard of care. There is limited knowledge about the patients’ perioperative experiences of bariatric surgery, with no study yet investigating their experiences within an opioid-free care pathway.

**Objective:**

We aimed to describe similarities and differences in patients’ perioperative experiences of undergoing bariatric surgery with either an opioid-free or opioid-based care pathway.

**Design:**

A qualitative interview study

**Setting:**

A strategic sample of patients enrolled in an ongoing randomized controlled trial investigating the effects of opioid-free anaesthesia for bariatric surgery were recruited. In the randomized controlled trial, participants were randomized to either opioid-based anaesthesia or opioid-free anaesthesia, including transcutaneous electrical nerve stimulation as primary postoperative pain management.

**Participants:**

Twenty patients were interviewed 3 months after surgery: 10 participants in the opioid-free group versus 10 in the opioid-based group.

**Methods:**

Semi-structured interviews were conducted between December 2020 and February 2022 and analysed with qualitative content analysis.

**Results:**

The analysis yielded four categories and 12 subcategories. In Category 1, participants shared *diverse emotions before surgery,* including *anticipation* of a healthier life, but also *apprehensions* and *feelings of failure*. In Category 2, describing *liminality of general anaesthesia*, there were similar descriptions of *struggling to remember* the anaesthesia induction and *struggling to surface* when recovering from anaesthesia. However, some participants in the opioid-free group shared descriptions of *struggling to keep control*, describing accentuated memories of the anaesthesia induction. Category 3, *managing your pain,* showed similar *experiences and strategies* but different narrations of pain management, with the opioid-free group stating that *transcutaneous electrical nerve stimulation works but not when it really hurts*, and the opioid-based group describing *confidence in but awareness of opioids*. Throughout the overall perioperative time period, participants acknowledged Category 4, a *patient-professional* presence, stating that *preparations boost the feeling of confidence* before surgery and that they felt *confidence in a vulnerable situation* although *vulnerability challenges communication*.

**Conclusions:**

We highlighted the overall similarities in perioperative experiences of patients undergoing bariatric surgery. However, the differences in experiences during opioid-free anaesthesia induction need to be addressed in further implementation and research studies investigating strategies to reduce the sense of loss of control. More research is needed to facilitate the implementation of opioid-free treatment strategies into clinical practice and improve the patient care experience.

**What is already known about the topic**

• The perioperative care pathway for patients undergoing bariatric surgery may affect the patient's outcome due to increased risk of opioid-related side effects.
• Opioid-free anesthesia could enhance postoperative recovery without compromising pain control for patients undergoing bariatric surgery.

Alt-text: Unlabelled box

**What this paper adds**

• We found similarities in patients’ perioperative experiences, regardless of opioid-free or opioid-based anaesthesia for bariatric surgery.
• The differences in patients’ experiences during the anaesthesia induction phase underscore the need for personalized care to optimize opioid-free strategies and enhance patient experience.

Alt-text: Unlabelled box

## 1 Background

Obesity is a growing public health challenge worldwide and increases the risk of severe diseases, such as diabetes type 2 and cardiovascular diseases (World Health Organization, 2021). Bariatric surgery is an effective long-term weight loss intervention for individuals with severe obesity. It not only showcases a reduction in the risks associated with comorbidities, such as diabetes type 2, obstructive sleep apnoea, hyperlipidaemia, hypertension, cancer, and cardiovascular disease, but also contributes to a significant improvement in health-related quality of life (D'Hondt et al., 2011; Kolotkin et al., 2012; Singh et al., 2020; Sjöström et al., 2004; Sundbom et al., 2024). Internationally, the median age for patients undergoing bariatric surgery varies from 34 to 45 years, with 79 % being women (International Federation for the Surgery of Obesity and Metabolic Disorders, 2023). In order to perform bariatric surgery, general anaesthesia is required, and opioids are standard treatment for both general anaesthesia and postoperative pain management. However, researchers have shown that opioids may negatively impact the bariatric patient's early recovery, as it increases the risk of postoperative nausea and vomiting, respiratory depression, and excessive sedation (Forget, 2019; Stenberg et al., 2022).

If postoperative pain is not adequately addressed in the acute phase immediately after surgery, it can affect the patient's recovery process and persist in the late recovery stage. This is especially important regarding pre-operative pain and younger patients, as both factors have been shown to be predictors for severe postoperative pain after bariatric surgery (Hartwig et al., 2017; Raebel et al., 2013). Moreover, obesity is associated with increased risk of developing chronic pain and opioid misuse compared to the average population (Raebel et al., 2013). Thus, significantly more patients undergoing Roux-en-Y gastric by-pass surgery start using opioids within 24 months after surgery compared to population controls both with and without obesity (Svensson et al., 2022). The increased risk of opioid-induced side effects in this population underscores the necessity of implementing opioid-sparing strategies in the perioperative context (Stenberg et al., 2022).

Opioid-free anaesthesia is defined as a strategy whereby no opioids are used before and during surgery, whereas opioid-sparing analgesia avoids the use of opioids in the postoperative phase (Forget, 2019; Mulier, 2019). Previous quantitative studies indicate that opioid-free anaesthesia can improve the postoperative outcomes, especially the risk of postoperative nausea and vomiting, without compromising postoperative pain management or patient safety for several surgical interventions (Olausson et al., 2021). For bariatric surgery specifically, postoperative nausea and vomiting is the leading cause of readmission due to dehydration and should be prevented to enhance recovery after surgery (Kushner et al., 2020). Opioid-free anaesthesia may also improve the postoperative pain outcomes both immediately and at 24 h after bariatric surgery (Hung et al., 2022). In conjunction with opioid-free anaesthesia, the evidence supports adopting multimodal analgesia, promoting an overall opioid-sparing approach (Forget et al., 2023). Studies have shown that the non-pharmacological therapy, transcutaneous electrical nerve stimulation, provides effective postoperative pain control compared to intravenous opioids, reducing the need for postoperative opioids (Piasecki et al., 2023).

Despite extensive support from numerous quantitative studies regarding the feasibility of opioid-free anaesthesia and its enhanced outcomes after surgery, there remains a paucity of knowledge regarding the patient's perioperative experiences. The necessity of implementing opioid-sparing strategies for bariatric surgery is well grounded and with a high level of evidence supported by the Enhanced Recovery After Bariatric Surgery guidelines (Stenberg et al., 2022). To date, there is to our knowledge only one qualitative study describing patients’ perioperative experiences of bariatric surgery (Forsberg et al., 2014) and none covering the experiences of opioid-free anaesthesia. Therefore, gaining a deeper understanding of patients’ perioperative experiences may contribute to improvement in quality of care and provide qualitative research insights complementing the existing quantitative research.

## 2 Methods

### 2.1 Aim

We aimed to describe similarities and differences in patients’ perioperative experiences of undergoing bariatric surgery with either an opioid-free or opioid-based care pathway.

### 2.2 Design

This study was designed as a qualitative interview study using a semi-structured interview guide with open-ended questions (Supplementary Material Supplement 1) and analysed using content analysis (Lundman and Graneheim, 2008).

### 2.3 Participants

A strategic sample of adult patients undergoing either laparoscopic gastric by-pass or laparoscopic sleeve gastrectomy and enrolled in the prospective, randomized, non-blinded, non-commercial multicentre study *Effects of an Opioid Sparing Care Pathway for Patients undergoing Obesity Surgery* (the randomized controlled trial, Clinical trial: NCT03756961) were eligible for inclusion. On the day of surgery, the patients were randomized to either opioid-based anaesthesia (control group) or opioid-free anaesthesia, including transcutaneous electrical nerve stimulation as primary postoperative pain management (intervention group). The detailed anaesthetic and postoperative pain management protocols for both the intervention group and control group are presented in Supplementary Material Supplement 2. After the surgery, the research team selected a strategic sample of patients based on their randomization group (intervention or control), study site, sex, and age. The research team identified 20 eligible patients during the data collection period (December 2020 to February 2022) and invited them to participate in the interview study, either at discharge from hospital after surgery or during a telephone follow-up in the randomized controlled trial at the 3-month follow-up. All the eligible participants agreed to participate.

### 2.4 Setting

The bariatric surgery was performed at two hospitals: one university hospital in the southwestern part of Sweden and one smaller hospital in the mid-south. The participants were admitted to the hospital the same day as surgery and stayed a couple of hours in the post-anaesthesia care unit, followed by one night in the surgical ward before discharge. In this study, the results were based on the participants’ perioperative experiences of undergoing bariatric surgery. The perioperative period includes the pre-, intra-, and postoperative phases. The preoperative phase captures the time from decision to have surgery to patient arrival in the operating room, including preparations before surgery, preoperative assessment, and hospital admission (Davrieux et al., 2019). Hence, the participants’ narratives encompassing the preoperative phase might have extended well before hospital admission. The following intraoperative phase defines the time in the operation room, starting with anaesthesia induction followed by performance of the surgical procedure, and ending with transport to the post-anaesthesia care unit (Edrees, 2024). The participants’ experiences during the intraoperative phase focused on the awake time in the operating room up to induction of general anaesthesia. The last postoperative phase captures the recovery from anaesthesia and surgery throughout the transition from the post-anaesthesia care unit to the surgical ward and may continue beyond hospital discharge (Davrieux et al., 2019; Edrees, 2024). However, the researchers chose to focus on the early postoperative experiences occurring in the post-anaesthesia care unit and surgical ward prior to hospital discharge, despite the postoperative phase extending beyond this point.

### 2.5 Data collection

The interviews were conducted by the authors AO and EA between December 2020 and February 2022. All interviews were conducted individually over the telephone, using speaker phone, and recorded with two separate recording devices. The participating patients gave their permission to record the interviews. The audio recordings were de-identified and coded with the participants’ enrolment numbers in the randomized controlled trial. All interviews were conducted in Swedish, using a semi-structured interview guide with open-ended questions concerning the patient's experience of perioperative in-hospital care, recovery after surgery, and postoperative pain up to 3 months after bariatric surgery (Supplementary Material Supplement 1). The collected data capturing the experiences after discharge from the hospital will be the subject of a future publication.

### 2.6 Data analysis

A qualitative content analysis with an initial inductive and subsequent deductive approach was employed to analyse the data (Graneheim et al., 2017; Lundman and Graneheim, 2008). The audio recordings from the interviews were transcribed by an external transcriber, and all transcripts were placed into a qualitative data analysis software (NVivo 12). To begin, the transcripts were read multiple times to gain a comprehensive understanding of the overall content. Then a primary analysis was executed after the first 13 interviews were conducted in late 2021, indicating saturation was not met, as new patterns emerged. In early 2022, seven more interviews were conducted, and new patterns no longer appeared. The data analysis process was primarily conducted by the first author AO and secondly by the author EA, supported by BH for triangulation between the three authors. In the final stage, the coding framework was discussed within the research group to reach consensus. All phases of the analysis process were conducted in the Swedish language.

#### 2.6.1 Inductive approach – first step

In the first step, the meaning units were abstracted, if necessary, and subsequently labelled with a code (Graneheim et al., 2017). While searching for overall patterns in the patients’ experiences of the perioperative care pathway, the codes were sorted into domains representing the pre-, intra- and postoperative phases. The codes were further structured into preliminary categories based on their commonalities; e.g., “positive” versus “negative” experiences.

#### 2.6.2 Deductive approach – second step

In the second step, the coding framework built on the perioperative timeline was subjected to subsequent deductive analysis, testing the implications of the pre-existing “positive” versus “negative” patterns for further investigation of differences and similarities between groups (Graneheim et al., 2017). When a deductive approach was employed, the initial categories yielded new dimensions to the analysis, moving away from the domain-based coding framework; e.g., *“struggling to keep control”,* instead of *“negative experiences of opioid-free anaesthesia”*.

### 2.7 Reflexivity

Data were collected and primarily analysed by the first AO and the second EA author. Both interviewers are native Swedish speakers, and the interviews were conducted in Swedish. AO is a doctoral student, nurse anaesthetist, and project coordinator in the randomized controlled trial with good knowledge in both the research field of opioid-free anaesthesia and the perioperative care pathway for patients undergoing obesity surgery. AO was familiar to most participants due to prior encounters both in-hospital and through telephone follow-up in the randomized controlled trial. The participants were consequently aware of the researcher's motivations for conducting this study. EA, in contrast, is a district nurse with a PhD and significant experience of qualitative research. BH, the third author who assisted the analysis, is a registered nurse with a PhD and senior researcher with expertise in qualitative research. Neither EA nor BH possesses specific expertise in the research field of opioid-free anaesthesia in obesity surgery. The other authors who supported and supervised the work have extensive knowledge in the research field of anaesthesia, perioperative pain medicine, and qualitative research methodology.

### 2.8 Ethical considerations

This qualitative interview study was part of a randomized controlled trial, which was approved by the ethical review board in Sweden (DNR 1006–17). Participating patients received both oral and written study information before signing the informed consent for the randomized controlled trial, which included the possibility of participating in the qualitative study. All participants were assured of privacy, anonymity, and confidentiality before giving their permission to record the interviews. The participants were also informed of their right to discontinue the interview at any time, due to the risk of evoking unpleasant or painful experiences from the in-hospital care. If any participants did not feel recovered at the time of interview 2–3 months after surgery, the interviewer would direct the participant to the bariatric clinic for further assessment.

## 3 Results

Twenty patients (14 women and six men) aged between 24 and 56 years old participated in the study. The median duration of interviews was 48.5 min (range: 26–70 min). The opioid-free anaesthesia group consisted of 10 participants, as did the opioid-based anaesthesia group likewise (Table 1). Four categories and 12 subcategories emerged from the qualitative content analysis, as presented in Table 2. The process of content analysis is presented in Table 3.

**Table 1:** Characteristics of study participants (*GBP = Gastric by-pass, **SG = Sleeve gastrectomy).

Participant ID	Randomization group	Sex	Age	Education level	Surgical procedure (GBP*/SG**)
1	Control	Male	40	High school	GBP
2	Intervention	Female	37	High school	GBP
3	Control	Female	24	University	SG
4	Control	Male	47	High school	SG
5	Control	Female	44	High school	SG
6	Intervention	Female	42	University	GBP
7	Control	Female	33	University	SG
8	Intervention	Female	41	High school	GBP
9	Control	Female	26	High school	GBP
10	Intervention	Female	35	High school	GBP
11	Intervention	Male	49	University	SG
12	Control	Female	32	High school	GBP
13	Control	Female	43	High school	GBP
14	Control	Male	50	University	GBP
15	Control	Female	31	University	GBP
16	Intervention	Male	52	High school	SG
17	Intervention	Female	34	High school	GBP
18	Intervention	Female	56	High school	GBP
19	Intervention	Female	34	Primary school	GBP
20	Intervention	Male	28	High school	GBP

**Table 2:** Outcome of the content analysis visualizing similarities and differences between groups. The ‘X’ symbol denotes instances of narrations within the respective subcategories for each group.

Category	Subcategory	Opioid-free group	Opioid-based group
Diverse emotions before surgery	Anticipation	X	X
	Apprehensions	X	X
	Feelings of failure	X	X
Liminality of general anaesthesia	Struggling to remember	X	X
	Struggling to keep control	X	
	Struggling to surface	X	X
Managing your pain	Experiences and strategies for managing pain	X	X
	Confidence but consciousness in opioids		X
	Transcutaneous electrical nerve stimulation works, but not when it really hurts	X	
Patient-professional presence	Preparations boost the feeling of confidence	X	X
	Confidence in a vulnerable situation	X	X
	Communication in pain treatment	X	X

**Table 3:** The process of content analysis.

Meaning unit	Code	Subcategory	Category
The [operating room] lamps felt like unidentified flying objects. I heard everyone talking but could not understand. I think I experienced hallucinations or being in another world.	Feelings of unreality	Struggling to keep control	Liminality of general anaesthesia

### 3.1 Diverse emotions before surgery

This category captured the participants’ journey before surgery, constituting descriptions of diverse emotions divided into the three subcategories: *anticipation, apprehensions*, and *feelings of failure.*

Participants shared *anticipation* for a healthier life, expecting the surgery to be a help on the way to weight reduction, reduced food intake, decreased risk for co-morbidities, more energy, and higher quality of life. However, they also described *apprehensions* before surgery, consisting of nervosity and fear. Nervosity was described on a general level, while fear was mainly focused on complications in relation to the anaesthesia, the surgical procedure, and the surgical outcome. Some participants were worried they would not achieve their weight loss goal before surgery and would therefore not be accepted for surgery. A commonly described fear was not waking up from anaesthesia or waking up in pain. Moreover, some participants dealt with long-term apprehensions; for example, not being able to eat like before and undergoing a major life-changing procedure. Some participants also described insufficient information, including information regarding the preoperative preparations, anaesthesia- and surgical procedure, as well as postoperative information regarding the recovery time in the post-anaesthesia care unit. Further, participants experienced *feelings of failure* when they could not manage their weight reduction on their own and had to give up after years of attempting to lose weight.

### 3.2 Liminality of general anaesthesia

This category emerged from the state of transition in-between wakefulness and sleep, capturing the time period of anaesthesia induction to emergence from anaesthesia. The category yielded three subcategories: *struggling to remember, struggling to keep control,* and *struggling to surface*.

*Struggling to remember* was a shared experience between groups, characterized by diminished memories of being anaesthetized. The participants mainly described few memories of being anaesthetized or described it as a very quick procedure and suddenly waking up in the post-anaesthesia care unit.

*“I didn't have that much time to think really. I had some questions there, but then, when everything was prepared and I got oxygen, I remember that I had an oxygen mask with 100 %. Then he said, you'll fall asleep soon, and that was true, it went really fast.”*

(Male participant, opioid-free anaesthesia, #11)

In both groups, a few participants with previous experience of being anaesthetized described the procedure as equal to past ones. For example, one participant receiving opioid-free anaesthesia described it as comparable to previous procedures.

*Struggling to keep control* was described by some participants in the opioid-free anaesthesia group. They described the anaesthesia induction procedure as a long journey to falling asleep, causing feelings of stress and panic when breathing oxygen (preoxygenation) through the mask. Some described accentuated memories of being put to sleep, of being between wakefulness and sleep, of feeling unable to get anywhere, and of experiencing a sense of unreality and attempting to struggle against it but then falling into sleep.

*”When these lamps hit my face, then it felt like, you know, a UFO. And that everyone was standing around me, I heard the people talking, but I couldn't understand what they said. Yes, I think I experienced hallucinations, in one way or another, or being in another world.”*

(Female participant, opioid-free anaesthesia, #2)

*Struggling to surface* included various experiences merging from early recovery in the post-anaesthesia care unit, with the groups describing similar things. Most commonly, the participants shared experiences of nausea, and, in a few cases, vomiting. The participants also described feelings of being very tired and dizzy. Some participants described unclear memories after waking up, remembering only their early recovery from anaesthesia or sleeping throughout their post-anaesthesia care unit stay. The tiredness was also described in terms of being able to hear but not open their eyes or lack of energy in mobilization. When waking up after anaesthesia, some participants experienced stress and, in a few cases, feelings of panic. They described these feelings as being caused by worrying over the surgical outcome, visual illusions, or a noisy atmosphere.

*“I felt panic, and it felt heavy. It felt like I was heavily sedated in some way. I felt like I almost, I don't know how to describe it, it felt like I was underneath 20 mattresses and needed to struggle myself up. It felt like I needed to force myself to wake up. I had to fight, because I heard, like, I needed to struggle myself to consciousness. I thought it was really hard to wake up and there were so many people and a lot of talking and noise, so that was a stress factor, it really was.”*

(Female participant, opioid-free anaesthesia, #10)

However, in both groups, a few participants described a general feeling of well-being when waking up from anaesthesia, experiencing few or no side effects.

### 3.3 Managing your pain

This category focuses on the postoperative pain experience, including attitudes and strategies for managing the pain during the in-hospital time period. The category generated three subcategories: *experiences and strategies, confidence in but awareness of opioids,* and *transcutaneous electrical nerve stimulation works, but not when it really hurts*.

*Experiences and strategies* were the core items conceptualizing postoperative pain management. In both groups, the majority experienced postoperative pain located in the abdomen, chest, oesophagus, and shoulders. Pain intensity varied from discomfort to severe postoperative pain, irrespective of whether the anaesthesia was opioid-free or not. However, in both groups, some participants recollected no pain at all. The participants suffering from severe postoperative pain in the post-anaesthesia care unit described the pain as being a cut in the stomach, having barbed wire or needles in the oesophagus, or as if all analgesia were suddenly gone.

*“Waking up in the post-anaesthesia care unit, it was one of the worst experiences I have ever gone through in my life. Because the anaesthesia just stopped working, all pain relief was suddenly gone, like boom, and the pain I felt in my stomach was not visible pain but coming from the inside. It felt like someone was doing it [the operation] right then, without anaesthesia. That feeling. And it was extremely painful”*

(Female participant, opioid-based anaesthesia, #5)

In the surgical ward, similar experiences of postoperative pain were described, ranging from manageable to severe in both groups. Notably for this period, the participants suffering from severe pain commonly described it as deriving from laparoscopic gas, causing more pain in the shoulders than in the stomach. This shoulder pain was described as more long-lasting and distracted from the pain in the stomach. Some of those describing severe pain also felt that it was hard to cope with the situation due to uncertainty about how severe the pain would become and when it would subside.

Some participants described trusting one's body and understanding the pain as something natural and expected caused by the surgery and not necessarily as something dangerous.

*“Now that I had done the surgery and I experienced pain afterwards, it was just like, you realize, it just goes, it turns out just fine. The pain isn't dangerous, it doesn't necessarily need to be dangerous.”*

(Female participant, opioid-free anaesthesia, #19)

In both groups, the participants shared multiple experiences of taking control of their pain using self-care management strategies. The most described strategy to deal with the postoperative pain was being active and walking around to get rid of gas from the abdomen and recover faster. Other described strategies were to use distracting activities, to find pain-relieving angles and positions, to try massage, to emit gas, or simply to endure the pain.

*“I had certain positions when I sat or stood up. Well, there were also times when I just couldn't control it. It didn't matter what I did, it just hurt all the time, no matter what I did. You stood up, you laid down, you sat, you walked. There weren't many of those times, but sometimes in the ward, it just didn't matter what I did. And then I just chose to walk, walk, and walk.”*

(Male participant, opioid-based anaesthesia, #14)

*Confidence in but awareness of opioids* was a theme permeating some of the participants’ stories in the opioid-based group, describing opioids not only as pain relief but as a way to relax or ease stress and as a prophylaxis of pain breakthrough at night. However, the participants described a reluctance to use opioids out of fear for the drug or previous experiences of side effects.

*Transcutaneous electrical nerve stimulation works, but not when it really hurts* was the essence of the participants’ description in the opioid-free group using this method as their primary pain treatment strategy after surgery. Most participants who considered transcutaneous electrical nerve stimulation treatment as effective described the pain intensity as light to moderate. Some participants described the pain-relieving effect as a distraction, changing focus from the hurting stomach to the machine electrodes instead. The participants also appreciated the transcutaneous electrical nerve stimulation massage program, with some describing not having any actual pain but primarily using the treatment for comfort. Some participants said the transcutaneous electrical nerve stimulation treatment enhanced their autonomy by enabling self-management of their pain and pain treatment. Several also used the treatment as a prophylaxis in case of pain breakthrough, especially before going to sleep after surgery.

*“Yes, no, but I didn't have much pain. Once I thought it would be difficult, I took the TENS [transcutaneous electrical nerve stimulation] device. I used it quite frantically. And I actually used it the whole evening, I would say. Until I thought it almost burned the skin, then I felt that I should probably take a break. But otherwise I'm really impressed that I didn't feel more than this.”*

(Female participant, opioid-free anaesthesia, #2)

Some participants who described severe postoperative pain stated that their initial testing of the transcutaneous electrical nerve stimulation treatment gave insufficient relief and described it as being uncomfortable or even hurting, which led to an unwillingness to try it again. Some participants described the treatment as more painful than the original pain and that it added to their existing severe pain.

*“I have never tried TENS before. But damn, it was horrible. It might have worked better at a later stage. It felt like it was so new, and I think that if I had tried it again, although I never did, but if I had tried it like, the day after, well not even then. Maybe the week after it might have been more of a support. It really felt like I was being cut up”*

(Female participant, opioid-free anaesthesia, #17)

### 3.4 Patient-professional presence

This category captured the experiences of interaction between the patients and caregivers throughout the in-hospital period, yielding the subcategories: *preparations boost the feeling of confidence, confidence in a vulnerable situation,* and *vulnerability challenges communication.*

*Preparations boost the feeling of confidence* focused on participants’ experiences before surgery, when they commonly described being satisfied with the caregivers’ acknowledgment of their situation and agreed they were thoroughly informed about the pros and cons of bariatric surgery. The participants especially appreciated the written patient information and group lecture before surgery, which prepared them and contributed to calmness. They also highlighted that the pre-surgery information regarding postoperative pain management was helpful, especially concerning mobilization after surgery.

*Confidence in a vulnerable situation* emerged from feelings of security and comfort, which participants in both groups acknowledged the caregivers supplied throughout the perioperative care chain. They also pinpointed that a focused and respectful relationship enhanced their feelings of confidence and trust and that a human touch by the nurse anaesthetist decreased feelings of nervosity during the anaesthesia induction.

*“You saw everyone standing around, and yes, you were nervous, very nervous. But, then a nurse anaesthetist came forward, took my hand, told me that everything will be fine and really stroked my hand. And suddenly almost all my nervousness was gone. It was, it really was, what I needed then. Which is really nice. And then, then I fell asleep.”*

(Female participant, opioid-based anaesthesia, #9)

Some participants also appreciated a jocular atmosphere during the anaesthesia induction, which they said helped them relax and feel safe.

*“…Many people have asked, wasn't it scary to be put to sleep, no, not at all. The doctor, he made jokes and stuff, so I felt very relaxed actually.”*

(Female participant, opioid-free anaesthesia, #19)

*Vulnerability challenges communication* emerged from experiences of interaction with the caregivers in painful situations. In both groups, most participants shared descriptions of being acknowledged and participating in the pain treatment. Even though pain could not be controlled as wished for at times, they were still content with the support given by the caregivers. In contrast, a few participants in both groups experienced insufficient pain management, lack of understanding of their pain experience, and suffering.

### 3.5 Similarities and differences

All in all, the participants in both groups had predominantly similar experiences throughout the perioperative care chain. However, during the anaesthesia induction phase, half of the participants in the opioid-free group (*n* = 5) shared accentuated memories of *struggling to keep control* in contrast to nearly half of the opioid-based group (*n* = 4) and some participants in the opioid-free group (*n* = 3) sharing diminished memories of *struggling to remember*. Postoperatively, a few participants in both groups (opioid-free *n* = 2, opioid-based *n* = 1) shared experiences of stress and anxiousness when waking up, thus struggling to surface from anaesthesia. Nevertheless, in both groups, some participants (opioid-free *n* = 3, opioid-based *n* = 4) described a general well-being when waking up from anaesthesia. Further, the descriptions of postoperative pain experience were similar between groups, apart from the experience of pain treatment with transcutaneous electrical nerve stimulation, dependent on group allocation.

## 4 Discussion

In this qualitative interview study, we investigated patients’ experiences of undergoing bariatric surgery with either an opioid-free or opioid-based care pathway. From the findings, we illuminated not only the overarching similarities in experiences throughout the perioperative time period but also the nuanced differences during anaesthesia induction, which are addressed below. Most participants, regardless of group allocation, shared *diverse emotions before surgery*, similar descriptions of *struggling to remember* the anaesthesia induction, and in the early postoperative phase *struggling to surface* from anaesthesia. The postoperative pain experience, yielding the category *managing your pain*, elicited similar descriptions of pain intensity, localization, and duration, despite different primary postoperative analgesic regimens. For the whole perioperative time period, most participants acknowledged a *patient-professional presence* contributing to confidence in care. In this qualitative study, we have shed light not only on patients' experiences of opioid-free anaesthesia but also the lack of knowledge concerning perioperative experiences of bariatric surgery focusing on the anaesthesia procedure. We suggest that an opioid-free care pathway for bariatric surgery is feasible, with comparable experiences of the perioperative process. An opioid-free care pathway is warranted, particularly in light of the prevailing opioid crisis, where persistent opioid use after surgery is a contributor (Hah et al., 2017).

The identified differences in experiences during anaesthesia induction, causing accentuated memories for half of the opioid-free group and described in the subcategory *struggling to keep control*, need to be addressed. The participants’ narratives of being in a state between wakefulness and sleep and feelings of unreality is an important finding, possibly explained by the use of esketamine for anaesthesia induction in the opioid-free anaesthesia group. Esketamine is the S-enantiomer of the racemic drug ketamine, which itself is a mixture of the two enantiomers, R- and S-ketamine. Studies have indicated that high doses of ketamine may cause negative perioperative experiences, such as hallucinations (Avidan et al., 2017). A meta-analysis of Hung et al. (2022) exploring the impact of opioid-free anaesthesia on bariatric surgery reported psychomimetic adverse events described as hallucinations in two out of eight trials for which ketamine was used in their anaesthetic regimen. However, ketamine has not been observed to negatively impact patient safety and remains a valuable approach for perioperative pain control, particularly in individuals with high body mass index who are at high risk of opioid-related adverse effects, such as postoperative respiratory depression (Adegbola et al., 2023; Olausson et al., 2021). While esketamine and ketamine are related compounds, esketamine is associated with fewer psychomimetic adverse events (Xie et al., 2023) than ketamine. Despite this difference favouring esketamine as a better choice of N-Methyl-d-Aspartate antagonist in this regard, it is important to address the adverse effects attributed to ketamine when interpreting our findings, as the two drugs share a structural relationship. The mechanical discrepancy in drug administration between the intervention and control group is another aspect that may potentially play a role in the opioid-free patients’ description of the anaesthesia induction being a long journey to falling asleep, as the induction was initiated with a loading dose of dexmedetomidine five minutes prior to the bolus dose of esketamine. The bolus dose of esketamine was manually administered via the syringe pump and required a series of steps to accurately set and confirm the bolus dose, a process that involved multiple iterations due to default safety limits. In contrast, the control group received remifentanil using the target-controlled infusion technique, including an automatized initial loading dose and infusion rate based on a computerized pharmacokinetic model, eventually achieving a slightly shorter induction time in favour of the patient's comfort. However, many target-controlled infusion models lack data on obese patients and cannot therefore adequately predict the target concentration, hence increasing the risk of overdosing (Kim, 2021). In obese patients, the pathophysiological changes in the body affect both drug distribution and elimination, which have several implications for the patient's outcome and necessitate dosage adjustments. The obese patient's increased volume of distribution is associated with a decrease in drug concentration during the initial distribution phase after administration, suggesting an overall prolonged onset of the anaesthetic effect, which, theoretically, is another possible explanation in line with our findings (De Baerdemaeker et al., 2004; Ingrande and Lemmens, 2010; Kim, 2021).

The lack of consensus regarding recommended drug dosing and its optimal combinations needs to be addressed, as various opioid-free anaesthetic protocols and weight-based dosing scalars are employed within the research field of bariatric surgery (Hung et al., 2022). Considering the impact of obesity on the pharmacokinetics and pharmacodynamics of intravenous anaesthetic drugs, it is worth highlighting the conflicting applications of weight-based dosing scalars in published data, especially as each anaesthetic drug is subject to a certain dosing scalar. While studies within the context of opioid-free anaesthesia for bariatric surgery have predominantly described the utilization of ideal body weight (Feld et al., 2003; Ibrahim et al., 2022; Mansour et al., 2013; Mieszczański et al., 2023; Mulier et al., 2018), the Enhanced Recovery After Bariatric Surgery guidelines (Stenberg et al., 2022) advocate applying lean body weight for anaesthesia induction and total body weight for maintenance of anaesthesia infusion without further specification on which drug. The inappropriate application of weight-based dosing scalar for the bariatric patient may lead to either underdosing or overdosing, potentially impacting the patient's outcome. Hence, the lack of evidence-based opioid-free anaesthesia guidelines and the resulting uncertainty in precision of drug dosing underscores the importance of further research in order to minimize adverse effects and optimize patient safety and outcomes.

The identified differences in the patients’ experiences of the anaesthesia induction also highlighted the importance of comprehensive communication and emotional support in enhancing patient outcomes and experiences. Being anaesthetized and undergoing a surgical procedure means loss of self-control for the patient, but the experience can be mitigated through improved perioperative communication and emotional support from the perioperative nurse (Arakelian et al., 2017). Communication inadequacies may occur at various points in care but most often in transfer of care responsibility, and patients’ preoperative expectations may challenge their confidence in the perioperative care process if their expectations are unmet (Malley et al., 2015). Therefore, understanding the patient's vulnerabilities, acknowledging the patient's feelings, and providing information adapted to the situation may empower the patient and enhance the perioperative experience (Lekens et al., 2023). In the present study, participants described apprehensions related to anaesthesia, such as fear of not waking up from anaesthesia, which, according to Larsson et al. (2023), could indicate an underlying fear of surrendering self-control. Adequately addressing preoperative anxiety for bariatric surgery is of utmost importance, as it negatively impacts early postoperative pain outcomes and subsequent recovery after surgery (Gravani et al., 2020). Employing a preoperative knowledge exchange by eliciting the patient's narrative to comprehend anaesthesia-related apprehensions can facilitate generation of information about the opioid-free anaesthesia induction experience, thereby enhancing preparedness for surgery and preventing complications and prolonged hospitalization (Arakelian et al., 2017; Gravani et al., 2020).

Physical presence may also enhance the feeling of safety in the perioperative setting, which may be expressed with physical closeness (Larsson et al., 2023). Some participants in this study elucidated the feeling of *confidence in a vulnerable situation*, giving the example of human touch from the nurse anaesthetist during anaesthesia induction as helping to decrease patient nervousness. Moreover, maintaining patient autonomy and influence in care; e.g., by ensuring the patient's acceptance of holding the breathing mask closely over their nose and mouth during preoxygenation, might also reduce discomfort at anaesthesia induction (Sundqvist et al., 2018). Thus, being present in the moment and attentive to the patient's individual needs may facilitate partnership and enable participation in care. Conversely, the lack of presence challenges the patient's feeling of safety. If the anaesthesia providers are perceived as stressed or not physically proximal to the patient, feelings of insecurity may arise (Arakelian et al., 2017; Larsson et al., 2023). Indeed, participating in novel work tasks beyond the clinical routine, such as practising opioid-free anaesthesia within the context of this clinical trial, may lead to unintentional distancing in demanding situations. In another clinical trial, all key participants underwent a series of lectures and clinical training before independently practising opioid-free anaesthesia, which is probably a crucial aspect when adopting a new technique (Zhou et al., 2023). Practising opioid-free anaesthesia requires adequate knowledge and training (Forget et al., 2023), which in previous research has been shown to be lacking, and is subsequently a barrier to its implementation in clinical practice (Morrow et al., 2022; Velasco et al., 2019).

The similar experiences from the early postoperative recovery phase yielding the subcategory *struggling to surface* correspond well with those in the qualitative study by Forsberg et al. (2014), where the participants described being in a haze, feeling confused, wanting to sleep, and worrying about the surgical outcome when waking up in the post-anaesthesia care unit after gastric by-pass surgery. However, in the present study, a few participants in both groups expressed more pronounced descriptions of stress and panic when emerging from anaesthesia; e.g., experiencing visual illusions. This finding has the potential to be associated with emergence delirium, which may manifest as confusion and disorientation during the transition from unconsciousness to wakefulness after general anaesthesia, where postoperative pain, inhalational anaesthesia, and intraoperative use of benzodiazepines are possible risk factors coinciding with our study (Wei et al., 2021).

The experiences of acute postoperative pain was similar between groups despite different primary analgesic regimens. This finding is in line with a meta-analysis from 2021 (Olausson et al., 2021) investigating the effect of total opioid-free anaesthesia versus opioid-based anaesthesia, showing no significant difference for postoperative pain intensity between groups. However, the participants in the present study randomized to the opioid-free group received not only opioid-free anaesthesia intraoperatively but also transcutaneous electrical nerve stimulation treatment as primary rescue analgesia postoperatively, which differs from previously published randomized controlled trials included in the meta-analysis using opioids as rescue analgesia for all groups in the postoperative phase (Olausson et al., 2021). The subcategory *transcutaneous electrical nerve stimulation works, but not when it really hurts* suggests that the pain-relieving effect is more frequently reported by those participants describing light to moderate postoperative pain, whereas those describing severe postoperative pain experienced insufficient pain-relieving effect. Knowledge about transcutaneous electrical nerve stimulation treatment for postoperative pain following bariatric surgery is sparse. To our knowledge, there is only one published study in this specific area, indicating that it reduces postoperative pain compared to placebo after open bariatric surgery in adjunction to pharmacological pain management (Luchesa and Lopes, 2022). However, neither the specific transcutaneous electrical nerve stimulation intervention nor surgical technique is comparable to this study. Previous studies employing high-frequency, high-intensity transcutaneous electrical nerve stimulation corresponding to this study protocol demonstrated that it offers comparable analgesia to intravenous opioids for postoperative pain after gynaecological surgery and reduced postoperative opioid consumption (Piasecki et al., 2023). For those participants responding to transcutaneous electrical nerve stimulation treatment in this study, we found that it not only relieved postoperative pain but also enhanced patient autonomy and self-management of pain. Previous researchers have shown that, in addition to the pain-relieving effects of transcutaneous electrical nerve stimulation, its self-administration for postoperative pain following laparoscopic cholecystectomy is something patients feel confident about (Xu et al., 2020). This implies enhanced patient satisfaction and autonomy in pain management, encouraging the use of transcutaneous electrical nerve stimulation as a safe non-pharmacological alternative in an opioid-sparing care pathway. Nevertheless, the difference in response to transcutaneous electrical nerve stimulation treatment, indicating a correlation to pain intensity, is an interesting finding that emphasizes the need for further research focused on investigating the predictors of responsiveness to this treatment.

The attitudes related to pain management were also an interesting finding that needs to be addressed. In a qualitative study by Johnson et al. (2023) exploring the perceptions and behaviors of patients undergoing elective surgery in relation to opioid pain management, the respondents turned out to have conflicting intentions and opinions regarding postoperative opioid usage, despite being aware of the negative side effects. This was similar to the findings presented in our study in the subcategory *confidence in but awareness of opioids*. In contrast, the reluctance of some participants in the opioid-free group to reconsider transcutaneous electrical nerve stimulation after their initial negative experience in the post-anaesthesia care unit indicates a hesitant belief in the analgesic effect of this treatment. These findings highlight the need to elicit pre-existing beliefs before surgery and provide adequate information about postoperative pain management (Cho et al., 2021). Patient education and trial of transcutaneous electrical nerve stimulation in the preoperative phase could improve the postoperative experience (Ghaddaf et al., 2022), thus improving psychological resilience and preparedness and consequently facilitating pain control (Carr and Goudas, 1999).

In the findings, which underscore the importance of comprehensive communication, emotional support, and physical presence across all perioperative phases, we point to the necessity of a holistic care approach and the potential for person-centred care interventions. Person-centred care within the perioperative context encompasses understanding the patient as a unique individual and partner in care, implying a partnership accounting for the patient's own resources, needs, and beliefs (Arakelian et al., 2017). While the participants acknowledged a *patient-professional presence* contributing to *confidence in a vulnerable situation*, it was also noted that *vulnerability challenges communication*. The inevitable loss of self-control when undergoing surgery and transitioning through the liminal states of general anaesthesia challenge the patient's feeling of safety, hence demanding presence and participation in care based on the patient's individual needs (Larsson et al., 2023). The elements of a person-centred care approach are not only commendable but essential, as they may elevate the quality of care, foster the patient-professional partnership, and ultimately enhance the patient's satisfaction and recovery after surgery (Arakelian et al., 2017; Larsson et al., 2023).

### 4.1 Limitations

Given the severe impact of the COVID-19 pandemic, there were disruptions to the anaesthesia and surgical operations at our study sites, significantly reducing the number of patients undergoing bariatric surgery between 2021 and 2022. This led to limitations in the enrolment of study participants to the randomized controlled trial. Nevertheless, all the enrolled study participants who attended the 3-month follow-up in the randomized controlled trial and were invited to participate in this interview study willingly agreed to do so. The successful recruitment of participants to this interview study was likely due to the study population being familiar with the researchers from previous encounters within the clinical trial, and their presumable interest in the research topic (Negrin et al., 2022). However, it is important to acknowledge that participants’ familiarity with the researchers may introduce potential participant bias, including social desirability bias. It is plausible that such familiarity may have caused certain participants to consciously avoid or minimize negative information, while exaggerating positive or desirable aspects. Consequently, this participant bias could impact the precision and *reliability* of the study results (Bergen and Labonté, 2020). It is crucial to acknowledge the potential risk of recall bias in interviews conducted 3 months post-surgery, and a shorter recall period would have been preferable (Althubaiti, 2016). The decision to maintain the set time point for interviews aligned with our initial objective, which aimed to capture both perioperative and postoperative experiences up to 3 months after surgery. Due to the substantial volume of interview data, we have opted to present it in two separate manuscripts. Importantly, the postoperative experiences after discharge from the hospital will be explored in a forthcoming manuscript.

Regardless of the intention to perform a strategic sample ensuring equal distribution between the opioid-free and opioid-based participants, the stratified random sampling in the randomized controlled trial facilitated an equal sample during the data collection period. However, sex was skewed, with 70 % female participants, which could be seen as a limitation. The distribution is nevertheless consistent with previous research, showing that bariatric surgery is more common among women in Sweden (Stenberg et al., 2014) and discussed as a limitation in a similar Swedish qualitative study investigating the experiences of undergoing laparoscopic gastric bypass surgery (Forsberg et al., 2014). Disregarding the overrepresentation of women, we recruited a variety of ages and geographical distribution, possibly contributing to a richer variation in these phenomena.

In the data collection phase, there was a risk of inconsistency due to the primary analysis yielding unmet saturation and the demand for further data. The extensive data collection period may have been affected by the evolving process of the researchers acquiring new insights into the phenomenon that emerged during the primary analysis. These new insights might have influenced follow-up questions and narrowed the focus, hence compromising *dependability* (Graneheim and Lundman, 2004). Nevertheless, the initial patterns observed during the primary analysis gave us a foundation upon which to carefully address phenomena, such as different experiences in anaesthesia induction, as we conducted further interviews.

We note that our findings focusing on the early postoperative phase are concordant with the findings of Forsberg et al. (2014), thus facilitating the *transferability* of patients’ postoperative experiences of undergoing bariatric surgery to similar contexts, particularly as the present study comprised participants recruited from two study sites. The diversity of the sample and rich data enhanced *credibility*, along with the representations of quotations and transparency of the abstraction process, as illustrated in Table 3 (Graneheim and Lundman, 2004). However, as there are no previous qualitative studies describing patients’ perioperative experiences of undergoing an opioid-free care pathway (either in general or in the context of bariatric surgery), further qualitative research in the field is required.

## 5 Conclusion

Overall, the experiences of undergoing bariatric surgery were similar, regardless of whether the anaesthetic regimen was opioid-free or opioid-based, and rendered similar descriptions of the early postoperative recovery phase, including the pain experience. Given the ongoing global opioid crisis and increased risk of opioid-induced side effects for bariatric patients, we have highlighted the feasibility of adopting opioid-free alternatives into perioperative care. Differences in experiences emerging from the anaesthesia induction phase point to the need for quality of care improvements in opioid-free anaesthesia for bariatric surgery. This requires further research to investigate the opioid-free drug combinations tailored to the bariatric patient's needs and strategies to reduce the patient's perceived loss of control during the induction phase, thus improving the patient care experience.

## CRediT authorship contribution statement

**Alexander Olausson:** Writing – original draft, Visualization, Resources, Project administration, Methodology, Investigation, Formal analysis, Data curation, Conceptualization. **Eva Angelini:** Writing – review & editing, Methodology, Investigation, Formal analysis, Conceptualization. **Birgit Heckemann:** Writing – review & editing, Validation, Methodology, Formal analysis. **Paulin Andréll:** Writing – review & editing, Validation. **Pether Jildenstål:** Writing – review & editing, Validation. **Sven-Egron Thörn:** Writing – review & editing, Validation, Resources. **Axel Wolf:** Writing – review & editing, Validation, Supervision, Methodology, Conceptualization.

## Declaration of competing interest

The authors declare that they have no known competing financial interests or personal relationships that could have appeared to influence the work reported in this paper.

## Appendix Supplementary materials

Image, application 1

Image, application 2
