# Supplementary material for: Patients’ perioperative experiences of an opioid-free versus opioid-based care pathway for laparoscopic bariatric surgery: A qualitative study
Source: Int J Nurs Stud Adv. 2024 Apr 20;6:100201. doi: 10.1016/j.ijnsa.2024.100201 (PMC11080373; doi:10.1016/j.ijnsa.2024.100201)
Supplement: Supplementary file 1 [file mmc1.docx]

**Supplement 1: Interview guide**

**Theme: Experience of the care process**

1. I would like you to start by telling me what thoughts and concerns you had before surgery.
   1. Can you tell us more about any expectations?
   2. Can you tell us more about any fears?
2. Before the surgery, you came to the hospital for a preoperative assessment. I would like you to tell me more about how you experienced that visit.
   1. Did you feel that you received the information you needed before the operation?
   2. What more information and preparation would you have appreciated?
3. If we move on to talk about the in-hospital care when you underwent the surgery, could you tell us more about your experiences? You may start from the moment you arrive to the hospital and follow the whole care chain including the operation theatre, postanaesthesia care unit and finally the surgical ward until you were discharged.
   1. Can you tell us more about the experiences that you experienced more positively?
   2. Can you tell us more about the experiences that you experienced more negatively?
4. If we focus on the communication between you and the healthcare staff during the in-hospital care, could you tell us more about your experiences in that regard?
   1. For example, did you feel seen and listened to?
   2. Did you get the support you needed?

1. Is there any experience that you took with you from the in-hospital care, which you feel can help you better handle a similar situation in the future?
2. Is there anything you missed that you feel could have helped you better deal with the difficulties that may arise in connection to surgery?
3. How do you feel that Covid-19 has affected your surgery and the course of care afterwards?

**Theme: Pain**

1. Now, we will discuss pain in general, and you will be given some situation-based questions that I would like you to answer as comprehensively as possible. The purpose is for you to share more about your experience of pain and how you typically manage pain in your daily life.
   1. When you think of the word 'pain' - what thoughts and experiences come to mind for you?
   2. If you experience a minor physical trauma, for example, hitting your toe on a table edge, - how do you handle that pain?
   3. If you experience a somewhat more significant physical trauma, for example, falling off a bike and hitting your leg quite hard, - how do you manage that pain?
   4. If you are going to undergo a procedure that you know will be painful in advance, for example, having a tooth filled or extracted, how do you typically think and prepare for it? For instance, do you take pain relief before, after, or not at all?
2. Now, I'd like to focus on the pain specifically after your gastric bypass/gastric sleeve surgery. Can you tell me about your experiences regarding the postoperative pain?
   1. When you underwent this operation, you received pain relief according to an opioid-sparing/opioid-based program. Can you tell me how that worked for you?
   2. If you received PCA - can you tell me more about it?
   3. If you received TENS - can you tell me more about it?
   4. If you actively chose not to use TENS postoperatively - can you tell me more about that?
3. Would you recommend the pain management you received to others?
4. How did you perceive your ability to manage your pain after the operation? Did you, for example, use any strategies to manage the pain?
5. Did you feel that you received the information and support you needed to manage the pain?
6. How did you experience your involvement in the pain management?
7. Can you tell me more about how you communicated your pain to the healthcare personnel?
8. How did you perceive the tools used to assess your pain, such as the NRS/VAS scale? Did you find it useful; did it help you in any way?
