# Supplementary material for: Patients’ perioperative experiences of an opioid-free versus opioid-based care pathway for laparoscopic bariatric surgery: A qualitative study
Source: Int J Nurs Stud Adv. 2024 Apr 20;6:100201. doi: 10.1016/j.ijnsa.2024.100201 (PMC11080373; doi:10.1016/j.ijnsa.2024.100201)
Supplement: Supplementary file 2 [file mmc2.docx]

**Supplement 2: Anaesthetic and postoperative pain management protocols**

Routine monitoring was applied to all patients in the operation theatre. Preoxygenation was performed until end-tidal oxygen fraction >90 % and an automatic lung recruitment manoeuvre was applied after intubation. Patients in both groups received multimodal analgesia, i.e. paracetamol (1 g) and parecoxib (40 mg) intravenously (i.v.) after induction, and local infiltration anaesthesia at surgical site before skin incision and at skin close. All patients also received PONV prophylaxis, i.e. betamethasone (8 mg) and ondansetron (4 mg) i.v.

*Intervention group – opioid-free anaesthesia*

General anaesthesia was initiated with a continuous infusion of dexmedetomidine (0.2 μg/kg/h i.v.) followed by a loading dose of dexmedetomidine (16 μg i.v.) 5 minutes prior to induction. For induction, a continuous infusion of esketamine (0.2 mg/kg/h i.v.) was started followed by a bolus dose of esketamine (0.1 mg/kg i.v.), propofol (1.5-2 mg/kg i.v.) and rocuronium (0.6 mg/kg i.v.). After induction and intubation, inhalation of desflurane was started with minimum alveolar concentration (MAC) target 0.8 and adjusted according to bispectral index (BIS) 40-60, together with the continuous infusions of dexmedetomidine (0.2 μg/kg/h i.v.) and esketamine (0.1-0,3 mg/kg/h i.v.). A bolus dose of esketamine (0.1 mg/kg) was given at skin incision, skin closure and signs of stressful response, e.g. tachycardia and hypertonia. The medication dosages were calculated based on adjusted body weight (body length in cm – 100 = ideal body weight + 50 % of remaining excess weight). All patients received lidocaine (1 mg/kg i.v.) and midazolam (0.5 mg i.v.) before emergence from anaesthesia. In the post anaesthesia care unit (PACU), if the patient’s pain score was > 3 according to the numerical rating scale (NRS, 0 = no pain and 10 = the worst imaginable pain), the patient first received nurse-administered high-intensity, high-frequency (80 Hz) TENS up to 40 mA at surgical site for 1 min, and repeated once if inadequate analgesic effect (i.e. NRS ≥3). In case TENS had no effect, the patient then received a bolus dose of esketamine (0.1 mg/kg i.v.) followed by lidocaine (0,5 mg/kg i.v.). If none of the non-opioid pharmacological interventions had sufficient analgesic effect, the patient received oxycodone i.v. at the nurse’s discretion. Once the sedation level reached an acceptable state in the PACU, the patient was instructed on self-administration of TENS, with modulated pulse duration stimulation adjusted to an intensity chosen by the patient. In the surgical ward the patient continued both nurse- and self-administered TENS-treatment in addition to paracetamol (1 g every sixth hour) and parecoxib (40 mg every twelfth hour) with oxycodone i.v. as rescue.

*Control group – opioid-based anaesthesia*

General anaesthesia was initiated according to the department routine e.g. with a continuous i.v. infusion of remifentanil with target controlled infusion (TCI) technique. The target plasma concentration (CPT) was set to 6 ng/ml for induction, and approximately by effect-site concentration (CE) 3.2 ng/ml, propofol (1.5 – 2 mg/kg i.v.) and rocuronium (0.6 mg/kg i.v.) was administered. After induction and intubation, inhalation of desflurane was started with MAC target 0.6, and adjusted according to BIS (40-60), together with continuous infusion of remifentanil (4-10 ng/ml) dependent on nociceptive response. The medication dosages were calculated based on adjusted body weight. All patients received oxycodone (0.1 mg/kg i.v.), and clonidine (45+30 μg i.v). before emergence from anaesthesia. In the post anaesthesia care unit (PACU), if the patient’s pain score was > 3 according to NRS, the patient received oxycodone i.v. at the nurse’s discretion.
